# Effect of Bead Geometry and Layer Time on Microstructure and Thermomechanical Properties of Large-Format Polymer Composites

**DOI:** 10.3390/polym18010133

**Published:** 2026-01-01

**Authors:** Tyler M. Corum, Johnna C. O’Connell, Samuel Pankratz, Maximilian Heres, Jeff Foote, Chad E. Duty

**Affiliations:** 1 Mechanical and Aerospace Engineering Department, University of Tennessee, Knoxville, TN 37996, USAcduty@utk.edu (C.E.D.); 2 Loci Robotics, Inc., Knoxville, TN 37902, USA; 3 Manufacturing Science Division, Oak Ridge National Laboratory, Knoxville, TN 37932, USA

**Keywords:** additive manufacturing, polymer composites, material characterization, print parameters

## Abstract

Large-format additive manufacturing (LFAM) is a manufacturing process in which high volumes of material are extruded in a layer-by-layer fashion to create large structures with often complex geometries. The Loci-One system, operated and developed by Loci Robotics Inc., is an LFAM-type system that was used to print single-bead walls of 20% by weight carbon fiber reinforced acrylonitrile butadiene styrene (CF-ABS) using various print parameter inputs. This study observed the influence of bead width and layer time on thermomechanical performance via material characterization techniques that accounted for the complex microstructure of LFAM parts to develop a better understanding of parameter–structure–property relationships. Printed parts were characterized by measuring the coefficient of thermal expansion (CTE) and interlayer strength. Near the edges of the printed beads, microscopy revealed a “thinning effect” experienced by a shell composed primarily of highly oriented fiber as the bead width was increased; however, this effect was diminished with a higher shear rate. The CTE results demonstrated the influence of mesostructure on the thermomechanical response. Increased shear rates were expected to lower CTE in the x-direction due to a higher ratio of fiber oriented in the print direction, but this relationship was not always observed. For the larger bead widths printed at higher shear rates, the randomly oriented fiber at the core dominated the thermomechanical response and increased CTE overall in the x-direction. A heat transfer model was developed for this work to determine how much time was required for the deposited bead to cool to the glass transition temperature. Interlayer strength results revealed a rapid decrease once the printed layer time exceeded the time required for the extrudate to cool below the glass transition temperature.

## 1. Introduction

Additive manufacturing (AM) is a process in which material is extruded in a layer-by-layer fashion, adding material only where it is needed rather than removing unwanted material. Large-format additive manufacturing (LFAM) is a focus of AM that utilizes pelletized feedstock and single screw extrusion to create large (>1 m^3^) complex geometries at high deposition rates (~50 kg/h) [1,2,3,4]. The extruder travels along a print path (x-direction) while material is deposited in the form of a bead to create a single layer. With the completion of a layer, the next layer is deposited upon the previous layer perpendicular to the build surface (z-direction) until the desired build height is met. The LFAM process allows the manufacturing of large complex geometries, such as autoclave molds for the aerospace tooling industry, that are difficult to produce with traditional manufacturing techniques. The use of pellets in LFAM systems, rather than wound filament, allows the printing of large structures without requiring a heated build chamber, which results in cost savings as described in [5,6]. Fiber reinforced polymer (FRP) feedstock, commonly used for LFAM printing, increases part stiffness and decreases coefficient of thermal expansion (CTE) values [4,7,8,9]. The addition of fiber reinforcement material, however, introduces a high degree of anisotropy and creates a dependence of properties on the orientation of fiber reinforcement material within the LFAM structure [9,10,11,12,13]. The print parameters used during the manufacturing of these FRP parts dictate the structure and ultimately influence final properties.

Layer time is the time consumed to deposit a single layer and directly affects the strength between printed layers or “bond strength” due to the influence of temperature. Layer time can be dictated by part geometry, with linearly longer layers requiring more time to print, treated as an input during the manufacturing process, or calculated from the desired travel speed of the print extruder. Once extrusion begins and the material has been deposited, the high-temperature extrudate begins to naturally cool to the surrounding ambient temperature as well as the cooler sublayer. The time required for the extrudate to cool depends both on the temperature settings of the extruder and on the thermal mass of the extrudate. A larger thermal mass, determined by layer height and bead width, would typically require more time to cool. Once the extrudate cools to the glass transition temperature (*T_g_*), the deposited material changes at the molecular level from a rubbery to a more rigid glassy state. For stronger bonds between layers, the material should be deposited before the same location on the previous layer has cooled below *T_g_* [14,15,16]. Sun et al. [14] investigated bonding of small-scale AM parts via the cooling experienced by the extrudate and emphasized the influence of thermal history on final mechanical properties. Compton et al. [15] explored the influence of thermal strain on deformation in LFAM parts by recording the thermal history of a part during printing, and developed a predictive model to assist with parameter selection. Compton’s findings showed a direct relationship between extrudate temperature and deformation, with a need to keep the extrudate above *T_g_* until the next bead was deposited to reduce the likelihood of deformation or cracking between layers. The current work aims to specifically explore the relationship between layer time and extrudate temperature to better understand how interlayer bond strength may be limited due to insufficient layer bonding.

**Figure 1:**
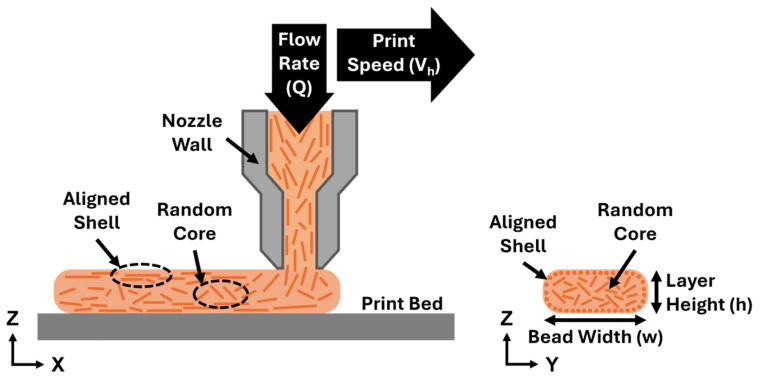
A diagram of LFAM extrusion, as well as the resulting arrangement of fiber due to shear forces at the nozzle wall.

The speed at which the extruder moves linearly across the build surface (print head speed or print speed) is set so that all material for the layer is deposited by the print head within the desired layer time. Print speed can either be set as an input or dictated by layer time and bead geometry. The geometry of the extruded bead is influenced by the volumetric flow rate, which is the rate of volumetric material that travels through the extruder nozzle. Print speed and volumetric flow rate are directly related. Increasing print speeds at a constant volumetric flow rate will result in smaller beads, while increasing volumetric flow rates at a constant print speed will result in larger beads. Volumetric flow rate (*Q*) can be set as an input directly or as a result of the desired print head speed (*v_h_*), bead width (*w*), and layer height (*h*), as shown by Equation (1). The volumetric flow rate directly influences the shear rate, which represents the frequency of interaction between the extruded material and the wall of the print nozzle. The average shear rate ($\dot{\gamma }$) experienced by the material is a function of Q and the radius of the extruder nozzle (*R*) as shown by Equation (2). A diagram of LFAM extrusion with these variables is shown in Figure 1 as well as the resulting arrangement of fiber due to the extrusion process. High shear rates represent more frequent interaction between fiber material and the nozzle wall during deposition that results in a higher amount of fiber oriented in the print direction. Because of this relationship, higher print speed results in fiber that is more oriented in the print direction.

(1) $$Q=v_{h}wh$$

(2) $$\dot{\gamma }=\frac{4Q}{{\pi R}^{3}}$$

As represented by shear rate, printing with FRP causes the fiber material to undergo an effect during extrusion referred to as fiber alignment, which occurs due to shear forces at the nozzle edge that align the fiber in the print direction. Fiber alignment results in a printed bead with fiber that is highly oriented (in the print direction) around the edge (shell) and randomly oriented fiber at the bead center (core) as a result of material extrusion, as shown by Figure 1 [17,18,19,20,21]. Sayah et al. [20] explored microstructural features that formed in LFAM parts due to the complex arrangement of fibers and observed this shell–core formation while examining micro-voids. Šeta et al. [21] modeled the distribution of fiber orientation within an LFAM bead and observed not only this shell–core trend but also that the simulated size of the core region increased at higher flow rates. The microstructure of printed FRP is nonhomogeneous due to fiber alignment since the degree of fiber orientation depends on proximity to the nozzle. This complex arrangement of fiber at the microstructural level further influences the thermomechanical response by creating a dependence, not only on fiber orientation, but also on location within the bead. This is referred to as mesostructure, which comprises the nonhomogeneous arrangement of microstructural features throughout the bead and layer. Due to both the complex mesostructure of LFAM parts and the anisotropic nature of the fiber material, it is difficult to accurately measure thermomechanical properties. Two-dimensional digital image correlation (DIC) has been shown as a viable technique to measure the strain experienced by a part by measuring displacement values across the sample surface [22,23,24,25,26]. Bing et al. [23,24] performed a review on DIC as a technique to measure surface deformation and provided guidance on experimental setup for testing CTE. Spencer et al. [26] demonstrated how DIC could be applied to FRP composite parts to reveal fiber orientation, which is relevant for LFAM parts given their complex microstructure. The current study utilized the DIC Oven, a characterization system developed at the University of Tennessee, to measure the thermal-induced distortion experienced by FRP printed via LFAM. This method allows a more complete representation of thermomechanical response for LFAM structures than traditional characterization techniques since the DIC Oven captures mesostructural characteristics [27,28,29]. Having this mesostructural level response is beneficial for LFAM parts when trying to correlate microstructural influences to print parameters.

This study specifically addressed how thermomechanical properties were influenced by LFAM print parameters, rather than mechanical properties alone. Understanding the thermomechanical performance of printed FRP is crucial for applications where LFAM parts will experience a range of temperatures, such as autoclave tooling. In this work, multiple bead widths were printed for each part to understand the impact of a larger bead width on the distribution of fiber orientation across the bead microstructure. Microscopy analysis was used to better understand the microstructure of the extrudate as influenced by shear rate, and the DIC Oven was used to capture the influence of these microstructural features on thermomechanical properties at a mesostructural level. A transient heat transfer model was developed to better understand the cooling experienced by each extrudate once material deposition was complete, and to compare cooling time to layer time. Mechanical testing revealed how layer bonding was impacted by different layer times because of bead cooling. Overall, this study aimed to characterize LFAM material at a mesostructural level, so the influence of print parameters could be correlated to final thermomechanical performance.

## 2. Materials and Methods

The material utilized in this study was 20% by weight carbon fiber reinforced acrylonitrile butadiene styrene (CF-ABS) supplied by Techmer Polymer Modifiers (Clinton, TN, USA). The Loci-One print system, custom-developed by Loci Robotics, was used to print each LFAM part. This system utilized a single screw extruder attached to the end of a KUKA 6-axis robot arm to achieve high precision and speed during printing. Each part was a single-bead thick wall that spanned only the x- and z-directions (XZ wall) with lengths of ~1.2 m and various inputs for both bead width and layer time. Microscopy analysis was performed to better understand the microstructure of the FRP printed beads. CTE testing showed the influence of nonhomogeneous microstructure on thermomechanical properties. A transient heat transfer model was developed in SolidWorks (Premium 2024 SP1.0) to understand the amount of time required for the extruded material to cool to *T_g_* based on the different thermal mass of each bead width. Mechanical testing was performed to show the influence of layer time on the interlayer strength.

### 2.1. Print Parameters

This study printed single-bead thick XZ walls of CF-ABS using a programmed layer time of 60, 120, or 240 s to simulate different-sized parts. Each part was deposited using either a continuous deposition (CD) or paused deposition (PD). For parts made using CD, the extruder deposited material during the entire programmed layer time at a print speed of 20, 10, or 5 mm/s. To achieve higher shear rates, PD parts were printed at 100 mm/s, and a dwell time (extruder idle) was introduced to maintain the same programmed layer time as their CD counterparts. A small (~7 mm), medium (~10 mm), and large (~14 mm) bead width were printed for each of these conditions using a constant layer height of 5 mm and a constant nozzle diameter of 5 mm. Volumetric flow rate was calculated from the programmed print speed, desired bead width, and 5 mm layer height. The shear rate experienced by each structure was determined from the resulting Q values. Parts were grouped 1–6 according to layer time and deposition technique, with a sample name used to identify individual parts. A summarized list of print parameter inputs, resulting Q and shear rates, and sample nomenclature is shown in Table 1.

### 2.2. Microscopy

Microscopy samples were prepared so the microstructure could be examined as a function of bead width and shear rate. Samples were sectioned using a IsoMet 1000 precision saw (Buehler, Lake Bluff, IL, USA), dried in a furnace overnight at 80 °C, and allowed to cool to room temperature in a desiccator. The samples were mounted such that the yz-plane was exposed in a 1.25-inch diameter puck using EpoxiCure2 (Buehler, Lake Bluff, IL, USA) and polished using an MetPrep 3 polisher (Allied High Tech Products, Inc., Cerritos, CA, USA). Imaging was performed for each sample using a VHX-5000 digital microscope (Keyence, Itasca, IL, USA).

Microscopy images were uploaded to ImageJ (version 1.54g) for processing, similar to the work described in [30]. The images were converted to gray scale so thresholding could be used to highlight different areas of fiber orientation. Thresholding was used so that approximately 97.5% of pixels were analyzed, with the upper bound pixel value set at 255 and the lower bound pixel value that was image dependent, based on brightness. This allowed the majority of background and highly oriented fibers (x-direction) to be removed from observation, while regions of randomly oriented fibers were revealed by their micro-voids. Yeole et al. [31] explained that these micro-voids occur along the fiber reinforcement material, and Sayah et al. [32] observed that higher concentrations of these micro-voids occurred towards the center of LFAM beads. Since the center of the bead is also where fiber is randomly oriented, these findings suggest that higher concentrations of these micro-voids can indicate areas of the bead where fiber is randomly oriented. The polygon tool was used to outline the region of the bead occupied by a randomly oriented fiber, and a measurement for area was calculated. The total area of the bead was calculated in the same manner using the polygon tool. This quantified the total bead area and the area occupied primarily by the randomly oriented fiber core. Subtracting these values provided the area of the bead occupied by the highly oriented fiber shell.

### 2.3. CTE Testing

**Figure 2:**
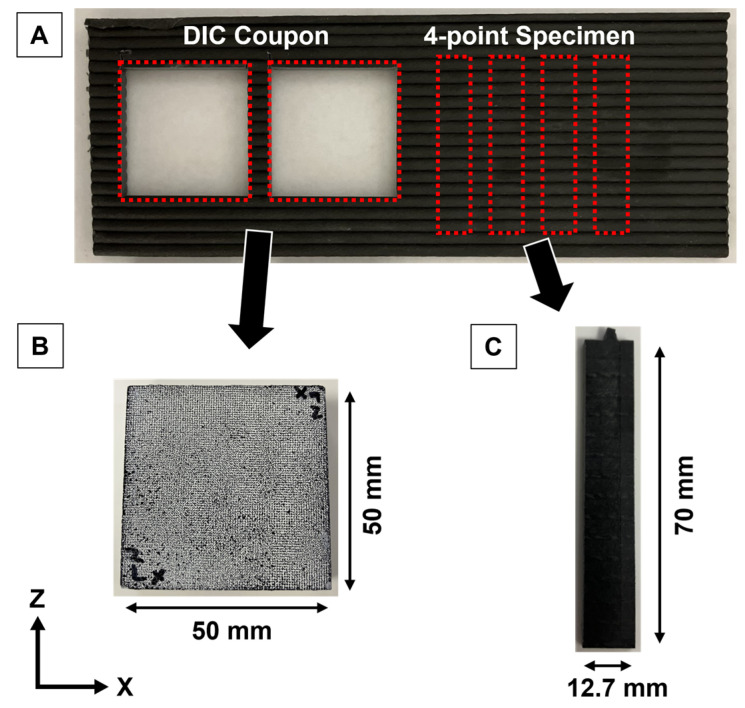
Sampling locations for DIC coupons and 4-point bend specimens from the printed XZ wall (**A**), a speckled DIC coupon (**B**), and a machined 4-point bend specimen (**C**).

Samples measured using the DIC Oven were prepared by milling a single side of each XZ wall until the beaded face was removed to ensure a flat and parallel face for accurate DIC testing [23,27,28]. A waterjet was used to cut two 50 mm × 50 mm test coupons from each XZ wall to better capture an average CTE value for each part, as shown in Figure 2. Each sample was dried in a furnace overnight at 80 °C and allowed to cool to room temperature in a desiccator. Samples were prepped for DIC testing as detailed in [28] by using high-temperature white spray paint and black ink to create a speckled contrast on the surface of interest, so that surface tracking could be used as described in [25,33].

DIC Oven testing was performed as described by the procedure in [27]. A set of images was taken at room temperature before the DIC Oven furnace was set to 90 °C, and the sample was allowed to reach a thermal equilibrium (steady state) for a period of at least 2 h before another set of images was taken at steady state. These images were uploaded to Correlated Solutions’ Vic-2D software (version 6.0.6) to calculate strain. The CTE for each sample was calculated, using Equation (3) below, for each sample based on the following known variables: strain at steady state (*ε_SS_*), strain at room temperature (*ε_RT_*), steady state temperature (*T_SS_*), and room temperature (*T_RT_*).

(3) $$\mathrm{CTE}=\frac{\varepsilon_{SS}-\varepsilon_{RT}}{T_{SS}-T_{RT}}$$

DIC Oven testing was performed using both a normal and a flipped orientation for each sample, as described in [27] to reduce bias from lighting or alignment effects. This procedure was completed twice for each sample to ensure consistency. The average CTE values that represent each sample comprised four total tests (normal and flipped orientation for two test coupons) that were averaged together based on corresponding x- and z-direction values.

### 2.4. Transient Heat Transfer Model

This study used SolidWorks to develop a 2D heat transfer model to quantify the time required for a deposited extrudate to cool to *T_g_*. The model consisted of representative beads that were based on the average bead width of the small (7.74 mm), medium (10.68 mm), and large (13.99 mm) bead sets. Each bead simulated in this model had a layer height of 5 mm to correspond to the printed parts of this study. This transient model spanned a total of 300 s across 2 s time steps as described in [34]. An initial temperature of 250 °C was applied to simulate deposition temperature. The heat transfer mechanisms applied to the model were conduction, convection, and radiation. Conduction from the newly deposited bead to the sublayer was accounted for by setting the bottom surface of the bead to *T_g_* with radiation and convection conditions applied to the remaining outer bead surfaces as described in [34]. Table 2 lists material property and heat transfer inputs used in the thermal simulation based on findings listed in [10,15] for Techmer CF-ABS. The mesh used in the model was identical to the solid high-quality solid mesh described in [34] and was solved using a blended curvature-based algorithm in SolidWorks. A maximum size of 0.32 mm was set for each triangular element within the mesh based on a sensitivity analysis of stable results. Once the transient simulation was complete, temperature data from nodes that comprised the top surface of the bead (where the next extrudate would be deposited or “surface of interest”) were recorded over time and averaged. This data was used to estimate surface temperature and plotted over time to determine the time required by each representative bead to cool from deposition temperature to *T_g_*.

### 2.5. Mechanical Testing

Samples measured using 4-point bend (4-pt) flexural testing were based on ASTM D6272-17 Procedure A described in [35] to quantify the influence of layer time on interlayer bonding of the printed samples. Each XZ wall was milled from both sides in order to achieve a final thickness of 3 mm from the most center portion of the wall. Four specimens were cut from each wall using a waterjet with final dimensions of 70 mm × 12.7 mm × 3 mm as shown in Figure 2. The sample specimens were dried in a furnace overnight at 80 °C and allowed to cool in a desiccator to room temperature before testing. A Universal Testing 5567 series load frame (Instron, Norwood, MA, USA) with a 30 kN load cell was used to test the interlayer bond strength. The span was set to 48 mm with a constant crosshead speed of 1.42 mm/min for each specimen. Loading was applied perpendicular (along the y-direction) to the XZ walls in a way that the load was experienced predominantly between layers without influence of x-direction properties, just as in [34]. The recorded strength for each specimen was averaged to represent a single sample.

## 3. Results and Discussion

The resulting microscopy analysis, CTE response, and interlayer strength are described for the printed XZ walls. Microscopy results showed effects of print parameters on the microstructure of the part, while DIC Oven and 4-pt testing captured effects of the print parameters on thermomechanical performance of the part at a mesostructural level.

### 3.1. Microscopy Results

**Figure 3:**
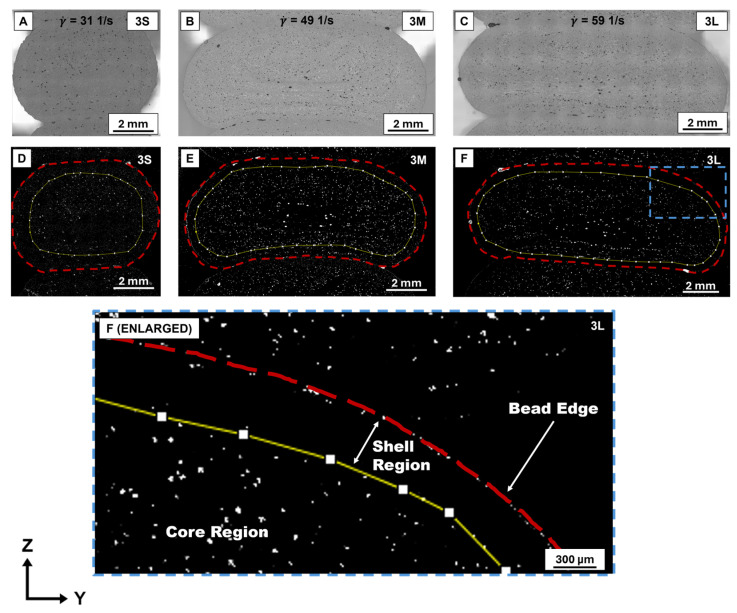
Microscopy images from sample group 3 (**A**–**C**) with the corresponding analyzed images (**D**–**F**) that show the distribution of fiber orientation for the small (**A**,**D**), medium (**B**,**E**), and large (**C**,**F**) bead width. Shown in the enlarged image (**F**), the yellow line represents the area occupied by a randomly oriented fiber with the red line outlining the total bead area.

The samples from group 3 (120 s layer time, 10 mm/s print speed, and continuous deposition) were chosen as a representative batch for the entire set of samples, given that they were printed using an intermediate layer time and print speed. Microscopy results are shown in Figure 3 for the different bead widths from group 3 (3S, 3M, and 3L). The microscopy images are shown in Figure 3A–C, with the ImageJ analyzed images in Figure 3D–F. For the images analyzed using ImageJ, the red dashed line represented the total cross-sectional area of the bead, and the yellow line represented the area occupied by randomly oriented fiber at the core region. The white areas in Figure 3D–F represent the micro-voids within the printed bead that revealed locations of randomly oriented fibers as described in Section 2.2. The area between the red and yellow lines represents the highly aligned shell region that resulted from the effects of fiber alignment, as shown by the enlarged version of Figure 3F. The constant nozzle diameter meant a higher Q was required to achieve a larger bead width, as indicated in Table 1. The constant layer height coupled with increased bead width required a higher material throughput, which caused the shell region to become thinner as bead width increased, which can be seen in the analyzed microscopy images. For the large ($\dot{\gamma }=\;$59 s^−1^) and medium ($\dot{\gamma }=\;$49 s^−1^) bead widths, there was a clear distinction between the shell and core; however, the distribution of fiber orientation for the small ($\dot{\gamma }=\;$31 s^−1^) bead width was more similar due to lower shear rate values.

To quantitatively compare the influence of increased bead width on microstructure, a ratio was calculated to represent the distribution of fiber orientation across each sample. This “core–shell ratio” normalized the area, with a higher core–shell ratio indicating a larger core relative to the shell. The shear rate, core area, shell area, and core–shell ratio for each sample in groups 3 and 4 are shown in Table 3. The core–shell ratio for group 3 increased rapidly with shear rate, indicating the highly aligned shell became thinner with increased bead width. From the small bead to the medium bead width, the core–shell ratio increased by 147% and then by 24% from the medium to large bead width. These results indicate that the highly aligned shell region decreased as bead width increased, which corresponds to the simulated results described by Šeta in [21]. These results indicate that for the group 3 samples, the region of the bead occupied by the aligned fiber decreased as bead width was increased, regardless of increased shear rate values. This signifies that bead width, not shear rate alone, was the dominant influence on the distribution of fiber orientation. When this process was repeated for the group 4 samples, however, the core–shell ratio appeared to be unaffected by increased shear rate. This was likely due to the higher overall shear rates experienced by the group 4 samples (~10× higher than group 3), which “stabilized” the impact of increased bead width on core–shell ratio.

### 3.2. DIC Oven Strain Plots

**Figure 4:**
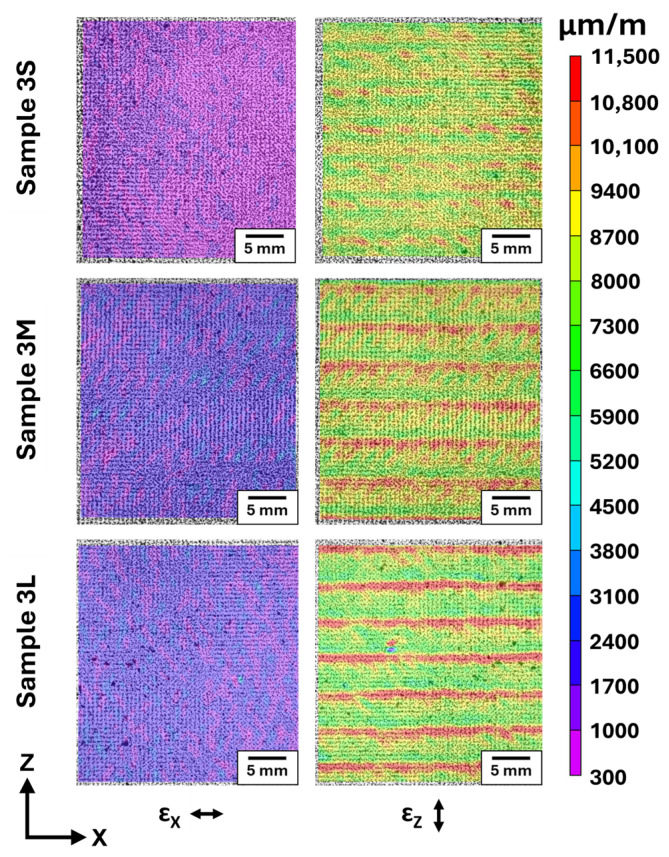
Contour plots of strain values created using Vic-2D show varying responses to thermal-induced strain based on varied bead width for sample group 3.

All images captured using the DIC Oven were uploaded to Vic-2D to create contour plots that represent the thermal-induced strain experienced by each sample. Representative strain contour plots that correspond to group 3 are shown in Figure 4. The strain plots showed that each bead width had a different thermomechanical response. For the x-direction strain (*ε*_x_) of each plot in Figure 4, there was a mostly homogeneous spread of relatively low strain (represented by purple-blue contour) due to fiber orientation within the bead. Since fibers were predominantly aligned in the x-direction via fiber alignment, the fiber reinforcement material provided more resistance to thermal expansion along the fiber (longitudinal direction), which resulted in lower *ε*_x_ overall. For the z-direction strain (*ε*_z_) plots in Figure 4, there were bands of high strain (red) that occurred at each layer interface throughout the XZ wall, with bands of lower (green-yellow) strain at each layer center. While the high strain was a result of anisotropic expansion across the aligned fiber, the prominent band effect was attributed to the nonhomogeneous microstructure of printed FRP material. As the bead width increased, the red high-strain areas at each bead interface became more defined. There were bands of high *ε*_z_ regions for sample 3S, but these bands were somewhat blurry relative to the low strain regions. For sample 3M, areas of high *ε*_z_ regions along the layer interfaces were more clearly defined compared to the lower strain at the layer center, and sample 3L showed well-defined areas of high *ε*_z_ regions along each layer interface. The more defined bands of high *ε*_z_ regions were attributed to the thinning shell region observed by the microscopy results of Section 3.1. To achieve a wider bead, it was necessary to increase Q, which increased shear rate, increased fiber alignment near the wall, and resulted in a more defined aligned shell. Once the samples from group 4 were processed in the DIC Oven, the high-strain interfaces between layers were approximately the same size for the 4S, 4M, and 4L samples, and each most resembled the effects from the sample 3L strain plot. This was expected based on the core–shell ratios for the group 4 samples from Table 3 and further supports the claim that the band effect observed by these strain plots was directly influenced by the distribution of fiber orientation within the printed bead.

### 3.3. DIC Oven CTE Testing

**Figure 5:**
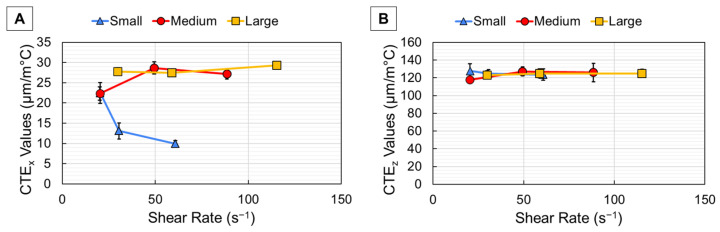
Resulting CTE values for both the x- (**A**) and z-direction (**B**) for samples printed using continuous deposition.

For continuous deposition (CD) printing, the average CTE results from DIC Oven testing are shown plotted with ± one standard deviation with respect to shear rate in Figure 5. Overall, the small bead width samples in Figure 5A showed that x-direction CTE (CTE_x_) decreased with increased shear rate due to higher fiber alignment, as expected. However, the medium bead width data in Figure 5A showed that the CTE_x_ values increased with higher shear rate, which was a non-typical thermomechanical response. This was indicative of the higher amount of randomly oriented fiber in the larger core section of the wider beads. This random trend is also apparent in the large bead samples of Figure 5. As bead width increased, the larger ratio of randomly oriented fiber at the core dominated the CTE_x_ response rather than the lower CTE_x_ provided by the highly aligned shell. This is effect supported by the findings in Table 3 and indicates that as bead width increased, the effect of shear rate was less pronounced, and CTE_x_ values increased as a result. The z-direction CTE (CTE_z_) values of Figure 5B showed that each bead width was relatively unaffected by the increasing shear rates. Since CTE_z_ values are primarily driven by high amounts of expansion that occurs transversely to the highly aligned shell, the constant layer height during printing kept the number of layer interfaces constant as bead width was increased, which resulted in CTE_z_ values that seemed unaffected by bead width. The CTE_z_ values of CF-ABS were ~10× higher than the measured CTE_x_ values due to the anisotropic nature of carbon fiber, with transverse CTE being approximately 10× higher than the longitudinal CTE [9,29,36,37]. The results for CD prints show that CTE values were primarily influenced by bead width rather than shear rate.

**Figure 6:**
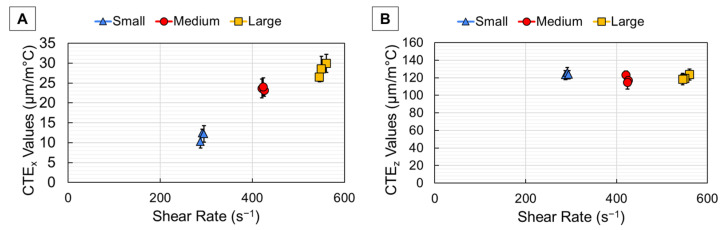
Resulting CTE values for both the x- (**A**) and z-direction (**B**) for samples printed using paused deposition.

For paused deposition (PD) printing, the average CTE results from DIC Oven testing are shown plotted with ± one standard deviation with respect to shear rate in Figure 6. Notice that for the PD prints, the shear rates are ~5× higher than the CD prints since a higher print speed (100 mm/s) was used for each sample. Based on the higher shear rates for the PD prints, it was expected that CTE_x_ would be lower than the CD prints due to higher fiber alignment, which occurred for both the small and medium bead widths of Figure 6A. However, the large bead width PD print did not exhibit much change from the CD print because there was only a slight difference in the core–shell ratio for the large bead, as shown in Table 3, which dictated thermomechanical response. Since the CTE_x_ data points were clustered together, it was evident that dwell time had little effect on thermomechanical response. As with the CD prints, the CTE_z_ values of Figure 6B did not increase with increased shear rate since the random core dominated thermomechanical behavior. This same trend of different dwell times clustered was true for the CTE_z_ values and indicated the lack of influence of dwell time on CTE. Since the CTE of these parts is mainly influenced by fiber orientation and not directly affected by layer time, it makes sense that both the CTE_x_ and CTE_z_ were not affected by varied dwell time.

### 3.4. Thermal Model Results

**Figure 7:**
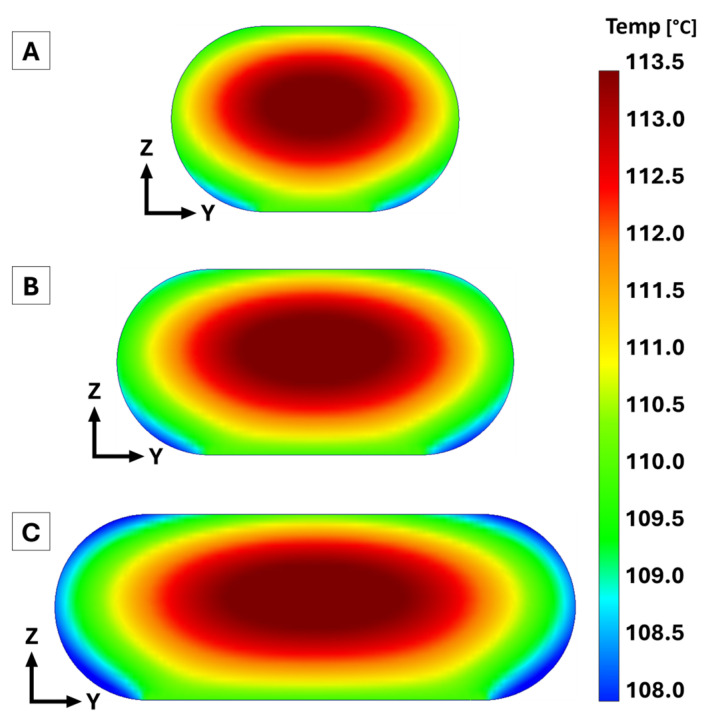
Cooling profiles from the transient heat transfer model for the small (**A**), medium (**B**), and large (**C**) representative bead as each bead cooled below *T_g_*.

**Figure 8:**
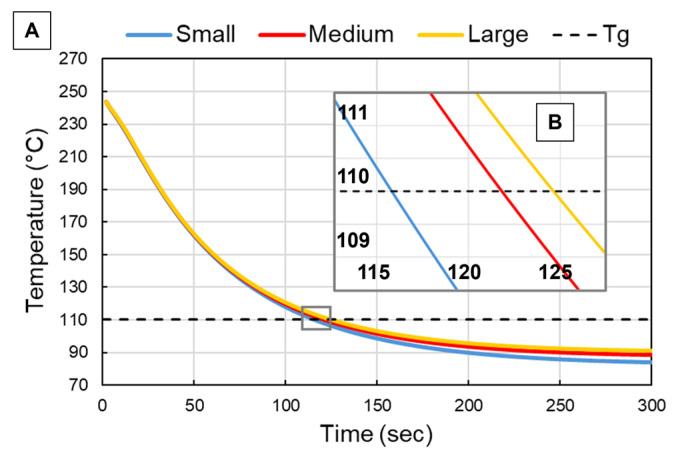
The cooling profile plotted from the transient heat transfer model for each representative bead (**A**) with an enlarged region (**B**) just after each bead cooled below *T_g_*.

The resulting cooling profiles for each bead in the transient heat transfer model are shown in Figure 7 as each bead cooled below *T_g_*. The overall pattern of the cooling profile featured concentric isotherms with a cool outer shell and a hotter center. The cooler outer shell was a result of heat escaping to the ambient environment via convection and radiation. Conduction was represented by heat escaping via the bottom surface to the previously deposited bead that had already cooled to *T_g_*. As bead width increased, so did the thermal mass (volume) of each extrudate; however, the surface area also increased. With increased surface area, more opportunity was provided for heat to escape from the surface of interest via convection and radiation. While larger bead widths did increase extrudate volume, the resulting increased surface area normalized the cooling of each bead overall, regardless of thermal mass. This was true specifically for this study since layer height was held constant in order to correspond to the printed beads of this study. If layer height had increased with bead width, this effect would not have been the case. The average temperature of the surface of interest for each representative bead is plotted in Figure 8A for each time step. Figure 8B shows an enlarged region to more clearly demonstrate how the different bead widths cooled below *T_g_*. Overall, there was a clear exponential decrease in surface temperature once the bead was deposited for each bead in the model. The small bead was the quickest to cool to *T_g_*, requiring approximately 116 s followed by the medium and large beads, which needed approximately 122 s and 126 s, respectively, to cool to *T_g_*. These simulated cooling results were similar to findings in [15], which monitored the layer-by-layer cooling of an LFAM part made of CF-ABS. Based on these results, the increased thermal mass did not drastically increase the time required for the extrudate to cool to *T_g_* since time values were within 8% of each other. While increased thermal mass did result in more required time to cool, increased bead width did not scale directly to increased cooling time (30% increased bead width vs. 8% increased cooling time) since the increased surface area normalized extrudate cooling.

### 3.5. Mechanical Testing Results

**Figure 9:**
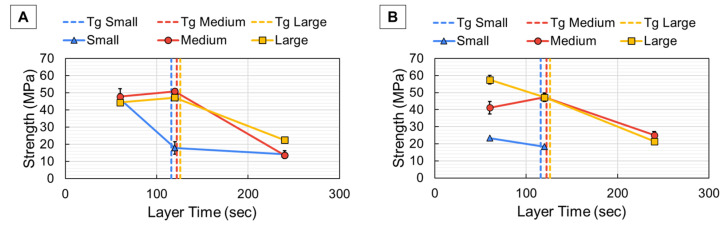
Results from 4pt-bend testing for the continuous prints (**A**) and the paused prints (**B**) with the time required for the bead to cool to *T_g_* represented by the dotted line.

The resulting interlayer bond strength (σ_z_) values from 4-pt testing are plotted with ± one standard deviation with respect to layer time in Figure 9. For the CD prints of Figure 9A, the strength between each layer ranged from 44 to 48 MPa for the 60 s layer time. The small bead width saw an immediate decrease in σ_z_ when layer time increased to 120 s, while medium and large beads maintained relatively consistent σ_z_ values at the 120 s layer time. However, once layer time increased to 240 s, the medium and large beads saw a rapid decrease in σ_z_ values. For the 240 s layer time, the strength recorded for each CD print ranged from 13 to 23 MPa, which signified a clear drop in σ_z_ values as layer time increased. The average time required by each bead to cool to *T_g_* is represented by the dotted lines on each plot of Figure 9. With the cooling data for each bead superimposed over the resulting strength, it was evident that the decrease in strength occurred once layer time exceeded the time needed for each bead to cool to *T_g_*. For the small bead of Figure 9A, which required approximately 116 s to cool to *T_g_*, there was a drop in σ_z_ for the 120 s layer time. The medium and large beads, which required approximately 122 and 126 s, respectively, to cool to *T_g_*, did not show a drop in σ_z_ until the 240 s layer time since neither had cooled to *T_g_* before the 120 s layer time. This trend of decreased σ_z_ values as layer time increased was true overall for the PD prints of Figure 9B. The PD prints had a much wider range of σ_z_ values for the 60 s layer time, spanning from 22 to 58 MPa. The reason for this difference in characteristic trends (specifically at the 60 s layer time) was the mechanism of failure. Since the PD prints were printed at higher overall print speeds, there was a higher shear rate and more defined fiber alignment, as supported by the findings in Section 3.1. Increased fiber alignment in the smaller bead produced a smooth, fiber-rich surface, which caused the break to occur along the layer interface where there was minimal engagement with the matrix. In contrast, there was less fiber alignment in the large beads, which allowed additional matrix bonding across the interface and resulted in a more tortuous fracture surface. This resulted in higher overall strength for the large bead width at the 60 s layer time, given the strength of the matrix material compared to the transverse strength of the fiber [8]. The medium bead experienced a combination of these effects, which fell between the low strength of the small bead and the high strength of the large bead. Overall, however, there was still a trend of weaker bond strength once layer time exceeded the time required to cool below *T_g_* for the PD prints. The strength of the PD parts after the 240 s layer time was very similar to their CD counterparts. The overall decreased σ_z_ values for these parts were expected based on the influence of *T_g_* on bond strength [14,15,16] and indicate the importance of understanding extrudate cooling so part strength does not diminish as a result of inadequate layer times.

### 3.6. Further Implications

This work explored the thermomechanical response for CF-ABS material printed via LFAM, but the overall trends from this study are applicable for the deposition of other shear-thinning fiber reinforced thermo-plastic polymers. Carbon fiber is routinely used in the aerospace industry due to a high stiffness-to-weight ratio; however, other fiber reinforcements like glass or Kevlar that have higher CTE properties and lower thermal conductivity will exhibit similar overall trends because they are primarily the result of fiber alignment. A variation in the amount of reinforcing content will influence these findings. Not only will higher amounts of fiber reinforcement increase the viscosity of the material (affecting printability), but additional reinforcement will generally improve the overall thermophysical performance of the material (lower CTE and higher strength).

## 4. Conclusions

This study characterized the thermomechanical response of LFAM printed CF-ABS using varied inputs for bead width and layer time. Microscopy was performed to better understand the effect of print parameters on the microstructure, and images were analyzed to quantify the distribution of fiber orientation. For slow print speeds (10 mm/s), it was found that as bead width increased, the highly aligned shell region of the bead became thinner relative to the randomly oriented core. The mechanism for the thinning shell was explained by an increased volumetric throughput (Q) at a constant nozzle diameter and layer height, which caused the bead to stretch. For high print speeds (100 mm/s), fiber orientation distribution appeared unaffected by increased shear rates due to higher overall shear rates that were independent of bead width.

The DIC Oven was used to capture the effects of fiber alignment on the thermomechanical response of each sample. The strain plots from the DIC Oven showed distinct high-strain regions at interfaces between layers due to expansion transverse to regions of highly oriented fiber. These areas of high strain became more defined as bead width increased. Given that shear rate increased to achieve these larger bead widths, the resulting increased fiber alignment resulted in more distinct regions of aligned fiber and more distinct changes in strain orthogonal to the layer interfaces. These effects were correlated with the thinning shell effect observed in the microscopy results.

The CTE values measured by the DIC Oven showed the influence of shear rate on CTE according to different bead widths and deposition methods. For the CD prints, the small bead width showed a decrease in CTE_x_ with increased shear rate due to higher fiber alignment. However, for the medium and large bead widths, CTE_x_ was relatively unaffected by increased shear rates since the CTE response was dominated by the randomly oriented core. For the PD prints, the CTE_x_ decreased overall from the CD prints for both the small and medium bead widths due to the higher print speeds, which increased shear rate and fiber alignment, but the large bead width was relatively unaffected since there was minimal change in the core–shell ratio from the CD print. For both CD and PD prints, the CTE_z_ values were relatively unaffected by increased shear rate since the expansion was dominated by the random core rather than the aligned shell. The layer deposition method did not appear to influence CTE based on the clustered data from the PD prints. The CTE values measured in this study show the influence of bead width on thermomechanical response and how mesostructure influenced final properties.

Mechanical testing revealed the influence of layer time on interlayer bonding, with supporting evidence from results of the transient heat transfer model. Overall, there was a drop in strength between layers once layer time exceeded the time required for the bead to cool to *T_g_*, which was consistent with previous work. The heat transfer model developed for this study illustrated the dependence of cooling behavior on print parameters such as bead width.

This study observed that bead width influenced CTE response by altering the distribution of fiber orientation within the LFAM bead, and interlayer strength decreased if layer time exceeded the time required for the extrudate to cool from deposition to *T_g_*. Future work may explore the introduction of a heat element to the deposition head to better regulate bead temperature so as not to limit bond strength based on layer time and part geometry. Findings from this work may also be used to better inform decisions for print parameters to prescribe a preferred microstructure in order to achieve more desired performance for LFAM parts.

## Figures and Tables

**Table 1:** Print parameters used in this study by the Loci-One system.

Layer Time [s]	Layer Deposition Method	Print Speed [mm/s]	Dwell Time [s]	Bead Width	Flow Rate [mm^3^/s]	Shear Rate [s^−1^]	Sample
60	Continuous	20	0	Small	746	61	1S
				Medium	1086	89	1M
				Large	1416	115	1L
	Paused	100	48	Small	3530	288	2S
				Medium	5162	421	2M
				Large	6882	561	2L
120	Continuous	10	0	Small	375	31	3S
				Medium	607	49	3M
				Large	722	59	3L
	Paused	100	108	Small	3578	292	4S
				Medium	5225	426	4M
				Large	6750	550	4L
240	Continuous	5	0	Small	250	20	5M1 *
				Medium	248	20	5M2 *
				Large	367	30	5L
	Paused	100	228	Small	3620	295	6S
				Medium	5203	424	6M
				Large	6697	546	6L

* Note: the group 5 samples had two similar widths that resembled values more like two medium-sized beads rather than a small and medium bead width.

**Table 2:** Input parameters for thermal analysis.

Variable [Units]	Value
Density, *p* [kg/m^3^]	1140
Specific heat capacity, *C* [J/kg-K]	1640
Thermal conductivity, *k* [W/m-K]	Dependent based on [10]
Natural convection coefficient, *h* [W/m^2^-K]	8.5
Emissivity, *ɛ* [---]	0.87
Glass transition temperature, *T_g_* [°C]	110
Deposition temperature, *T_dep_* [°C]	250
Ambient temperature, *T_∞_* [°C]	25
Layer height, *h* [mm]	5
Analyzed bead length, *l* [mm]	1
Bead width, *w* [mm]	7.74, 10.68, or 13.99

**Table 3:** Bead areas quantifying regions of fiber orientation as measured using ImageJ.

Sample	Shear Rate [s^−1^]	Core Region [mm^2^]	Shell Region [mm^2^]	Core–Shell Ratio
3S	31	18.6	12.6	1.5
3M	49	41.2	11.3	3.7
3L	59	51.1	11.3	4.5
4S	292	23.0	5.2	3.8
4M	426	35.7	7.5	4.8
4L	550	47.8	10.2	4.7

## Data Availability

The raw data supporting the conclusions of this article will be made available by the authors on request.

## References

[1] Duty C.E., Kunc V., Compton B., Post B., Erdman D., Smith R., Lind R., Lloyd P., Love L. (2017). Structure and Mechanical Behavior of Big Area Additive Manufacturing (BAAM) Materials. Rapid Prototyp. J..

[2] Vicente C.M.S., Sardinha M., Reis L., Ribeiro A., Leite M. (2023). Large-Format Additive Manufacturing of Polymer Extrusion-Based Deposition Systems: Review and Applications. Prog. Addit. Manuf..

[3] Sánchez D.M., De La Mata M., Delgado F.J., Casal V., Molina S.I. (2020). Development of Carbon Fiber Acrylonitrile Styrene Acrylate Composite for Large Format Additive Manufacturing. Mater. Des..

[4] Duty C.E., Drye T., Franc A. (2015). Material Development for Tooling Applications Using Big Area Additive Manufacturing (BAAM).

[5] Post B.K., Lind R.F., Lloyd P.D., Kunc V., Linhal J.M., Love L.J. The Economics of Big Area Additive Manufacturing. Proceedings of the 26th Annual International Solid Freeform Fabrication Symposium—An Additive Manufacturing Conference.

[6] Post B., Richardson B., Lind R., Love L.J., Lloyd P., Kunc V., Rhyne B.J., Roschli A., Hannan J., Nolet S. Big area additive manufacturing application in wind turbine molds. Proceedings of the 28th Annual International Solid Freeform Fabrication Symposium—An Additive Manufacturing Conference.

[7] Wang Z., Smith D.E. (2019). Numerical Analysis of Screw Swirling Effects on Fiber Orientation in Large Area Additive Manufacturing Polymer Composite Deposition. Compos. Part B Eng..

[8] Mallick P.K. (2008). Fiber-Reinforced Composites: Materials, Manufacturing, and Design.

[9] Love L.J., Kunc V., Rios O., Duty C.E., Elliott A.M., Post B.K., Smith R.J., Blue C.A. (2014). The Importance of Carbon Fiber to Polymer Additive Manufacturing. J. Mater. Res..

[10] Hassen A.A., Dinwiddie R.B., Kim S., Tekinap H.L., Kumar V., Lindahl J., Yeole P., Duty C., Vaidya U., Wang H. (2022). Anisotropic Thermal Behavior of Extrusion-Based Large Scale Additively Manufactured Carbon-Fiber Reinforced Thermoplastic Structures. Polym. Compos..

[11] Wang Z., Fang Z., Xie Z., Smith D.E. (2022). A Review on Microstructural Formations of Discontinuous Fiber-Reinforced Polymer Composites Prepared via Material Extrusion Additive Manufacturing: Fiber Orientation, Fiber Attrition, and Micro-Voids Distribution. Polymers.

[12] Pibulchinda P., Barocio E., Favaloro A.J., Pipes R.B. (2023). Influence of Printing Conditions on the Extrudate Shape and Fiber Orientation in Extrusion Deposition Additive Manufacturing. Compos. Part B Eng..

[13] Markandan K., Lai C.Q. (2023). Fabrication, Properties and Applications of Polymer Composites Additively Manufactured with Filler Alignment Control: A Review. Compos. Part B Eng..

[14] Sun Q., Rizvi G.M., Bellehumeur C.T., Gu P. (2008). Effect of Processing Conditions on the Bonding Quality of FDM Polymer Filaments. Rapid Prototyp. J..

[15] Compton B.G., Post B.K., Duty C.E., Love L., Kunc V. (2017). Thermal Analysis of Additive Manufacturing of Large-Scale Thermoplastic Polymer Composites. Addit. Manuf..

[16] Seppala J.E., Hoon Han S., Hillgartner K.E., Davis C.S., Migler K.B. (2017). Weld Formation during Material Extrusion Additive Manufacturing. Soft Matter.

[17] Colon A.R., Kazmer D.O., Peterson A.M., Seppala J.E. (2023). Characterization of Die-Swell in Thermoplastic Material Extrusion. Addit. Manuf..

[18] Brenken B., Barocio E., Favaloro A., Kunc V., Pipes R.B. (2018). Fused Filament Fabrication of Fiber-Reinforced Polymers: A Review. Addit. Manuf..

[19] Kumar V., Alwekar S.P., Kunc V., Cakmak E., Kishore V., Smith T., Lindahl J., Vaidya U., Blue C., Theodore M. (2021). High-Performance Molded Composites Using Additively Manufactured Preforms with Controlled Fiber and Pore Morphology. Addit. Manuf..

[20] Sayah N., Smith D.E. (2024). Correlation of Microstructural Features within Short Carbon Fiber/ABS Manufactured via Large-Area Additive-Manufacturing Beads. J. Compos. Sci..

[21] Šeta B., Sandberg M., Brander M., Mollah M.T., Pokkalla D., Kumar V., Spangenberg J. (2023). Modeling Fiber Orientation and Strand Shape Morphology in Three-Dimensional Material Extrusion Additive Manufacturing. Compos. Part B Eng..

[22] Chu T.C., Ranson W.F., Sutton M.A. (1985). Applications of Digital-Image-Correlation Techniques to Experimental Mechanics. Exp. Mech..

[23] Bing P., Qian K., Xie H., Asundi A. (2009). Two-Dimensional Digital Image Correlation for in-Plane Displacement and Strain Measurement: A Review. Meas. Sci. Technol..

[24] Bing P., Hui-min X., Tao H., Asundi A. (2009). Measurement of Coefficient of Thermal Expansion of Films Using Digital Image Correlation Method. Polym. Test..

[25] Lyons J.S., Liu J., Sutton M.A. (1996). High-Temperature Deformation Measurements Using Digital-Image Correlation. Exp. Mech..

[26] Spencer R., Alwekar S., Jo E., Hassen A.A., Kim S., Vaidya U. (2022). Fiber Orientation Evaluation in Reinforced Composites Using Digital Image Correlation and Thermal Excitation. Compos. Part B Eng..

[27] Corum T., O’Connell J., Brackett J., Spencer R., Hassen A., Duty C. Characterizing the Thermal-Induced Distortion of Large-Scale Polymer Composite Printed Structures. Proceedings of the 33rd Annual International Solid Freeform Fabrication Symposium 2022—An Additive Manufacturing Conference.

[28] Corum T., O’Connell J., Heres M., Foote J., Duty C. Characterizing thermomechanical properties of large-format printed composite polymer structures. Proceedings of the 34th Annual International Solid Freeform Fabrication Symposium 2023—An Additive Manufacturing Conference.

[29] Corum T.M., O’Connell J.C., Brackett J.C., Hassen A.A., Duty C.E. (2024). Measuring Thermomechanical Response of Large-Format Printed Polymer Composite Structures via Digital Image Correlation. Addit. Manuf..

[30] Wang P.H., Sterkenburg R., Kim G., He Y.W. (2019). Investigating the Void Content, Fiber Content, and Fiber Orientation of 3D Printed Recycled Carbon Fiber. Key Eng. Mater..

[31] Yeole P., Hassen A.A., Kim S., Lindahl J., Kunc V., Franc A., Vaidya U. (2020). Mechanical Characterization of High-Temperature Carbon Fiber-Polyphenylene Sulfide Composites for Large Area Extrusion Deposition Additive Manufacturing. Addit. Manuf..

[32] Sayah N., Smith D.E. (2022). Effect of Process Parameters on Void Distribution, Volume Fraction, and Sphericity within the Bead Microstructure of Large-Area Additive Manufacturing Polymer Composites. Polymers.

[33] Bing P., Xie H., Wang Z., Qian K., Wang Z. (2008). Study on Subset Size Selection in Digital Image Correlation for Speckle Patterns. Opt. Express.

[34] Corum T., Heres M., Foote J., Duty C. Influence of Bead Width and Layer Time on the Interlayer Bonding of Large-Format Printed Polymer Composites. Proceedings of the 36th Annual International Solid Freeform Fabrication Symposium 2023—An Additive Manufacturing Conference.

[35] (2010). Test Method for Flexural Properties of Unreinforced and Reinforced Plastics and Electrical Insulating Materials by Four-Point Bending.

[36] Billah K.M.M., Lorenzana F.A.R., Martinez N.L., Wicker R.B., Espalin D. (2020). Thermomechanical Characterization of Short Carbon Fiber and Short Glass Fiber-Reinforced ABS Used in Large Format Additive Manufacturing. Addit. Manuf..

[37] Daniel I.M., Ishai O. (2006). Engineering Mechanics of Composite Materials.

